# Common mechanisms of pain and depression: are antidepressants also analgesics?

**DOI:** 10.3389/fnbeh.2014.00099

**Published:** 2014-03-25

**Authors:** Tereza Nekovarova, Anna Yamamotova, Karel Vales, Ales Stuchlik, Jitka Fricova, Richard Rokyta

**Affiliations:** ^1^Institute of Physiology, Academy of Sciences of the Czech RepublicPrague, Czech Republic; ^2^Department of Zoology, Ecology and Ethology Research Group, Faculty of Natural Science, Charles University in PraguePrague, Czech Republic; ^3^Department of Normal, Pathological and Clinical Physiology, Third Faculty of Medicine, Charles University in PraguePrague, Czech Republic; ^4^Department of Anesthesiology and Intensive Care Medicine, Pain Management Center, First Faculty of Medicine and General University Hospital, Charles University in PraguePrague, Czech Republic

**Keywords:** chronic pain, depression, antidepressants, default mode network, neuroplasticity, stress, cytokines

## Abstract

Neither pain, nor depression exist as independent phenomena *per se*, they are highly subjective inner states, formed by our brain and built on the bases of our experiences, cognition and emotions. Chronic pain is associated with changes in brain physiology and anatomy. It has been suggested that the neuronal activity underlying subjective perception of chronic pain may be divergent from the activity associated with acute pain. We will discuss the possible common pathophysiological mechanism of chronic pain and depression with respect to the default mode network of the brain, neuroplasticity and the effect of antidepressants on these two pathological conditions. The default mode network of the brain has an important role in the representation of introspective mental activities and therefore can be considered as a nodal point, common for both chronic pain and depression. Neuroplasticity which involves molecular, cellular and synaptic processes modifying connectivity between neurons and neuronal circuits can also be affected by pathological states such as chronic pain or depression. We suppose that pathogenesis of depression and chronic pain shares common negative neuroplastic changes in the central nervous system (CNS). The positive impact of antidepressants would result in a reduction of these pathological cellular/molecular processes and in the amelioration of symptoms, but it may also increase survival times and quality of life of patients with chronic cancer pain.

## Introduction

Chronic pain is a complex syndrome which affects thinking, mood and behavior, and it can gradually lead to complete psychological and social isolation of the patient; therefore it can have a significant impact on everyday human activities, limiting independence and significantly interfering with interpersonal relationships. Mechanisms underlying chronic pain are different from those of acute pain. Chronic pain could lead to permanent changes of brain structures and functions and these changes could affect also brain processes not directly connected with pain itself (Baliki et al., 2008).

Depression is a psychiatric disorder with various symptoms and is often accompanied by unexplained painful somatic symptoms. In patients with somatic symptoms, especially in primary care, depression is frequently overlooked. On the other hand, psychiatrists do not pay enough attention to somatic or pain symptoms in patients with depression. Depression can precede pain or pain can precede depression, forming a linked dyad sharing common mechanisms (Blackburn-Munro and Blackburn-Munro, 2001; Chou, 2007). As argued by Torta and Munari (2010), depression may reduce the pain threshold and sensitize pain perception. Conversely, chronic pain may lead to an altered emotional state and finally to depression.

The concept of pain has changed dramatically over the last 30 years. As knowledge and understanding of pain have increased, the concept has gradually morphed from a one-dimensional concept to a multi-dimensional concept. In 90th Melzack (1999, 2001) postulated the existence of a pain neuromatrix, which can be thought of as a genetically determined neural network that is significantly modulated by stress, affective and cognitive processes (Iannetti and Mouraux, 2010). The pain neuromatrix is an integration of sensory, interoceptive, affective, and cognitive components and the resulting experience of pain is always dependent on their interactions (Klossika et al., 2006; Wiech et al., 2008; Simons et al., 2014). Various studies particularly emphasize the relationship between pain and emotions (Rhudy, 2009; Kamping et al., 2013). In addition, pain is affected by endogenous opioids, and the endocrine, immune and autonomic systems (Blackburn-Munro and Blackburn-Munro, 2003; Chapman et al., 2008).

This network is constantly under the control of many other factors including the state of attention, anxiety, and expectation; previous experience, learning, personality traits, cultural effects, etc., play important roles. Therefore, we can rationally assume that neuroplastic and cognitive processes have critical roles as links to emotional processes associated with patient suffering.

We will summarize intersections between chronic pain and depression mechanisms at different levels of complexity, i.e., brain networks (default mode network), neurotransmitter systems and neuronal plasticity. We will demonstrate that both chronic pain and depression can lead to stable changes in brain structure and function and that these changes are manifested in the patient’s experience, emotion and cognition. There is high comorbidity between chronic pain and depression in severe disorders and there is also a clear link between chronic pain and depression at the level of experience. Chronic pain can induce depression and depression can manifest as pain. In this study we suggest that the association between depression and chronic pain is not just at the level of experience, there may also be a common neural substrate, which could be therapeutically manipulated to improve overall quality of life for patients with both conditions.

### Default mode network as a mechanism of self-reference, nodal point of depression and chronic pain

Neither pain, nor depression exists as an independent phenomenon *per se*; they are highly subjective inner states, created by our brain and formed by our experience, cognition and emotional arousal. The default mode network is a network of interacting brain regions and subsystems that show consistently greater activation during “resting” states compared to external, directed tasks (often referred as “task-induced deactivation”) (Shulman et al., 1997). The brain regions involved in these self-referential processes are inversely correlated with the fronto-parietal regions that are typically associated with cognition (Fox et al., 2005).

The default mode network consists of a set of regions in the cerebral cortex—medial prefrontal cortex, posterior cingulate cortex and connected ventral precuneus, medial temporal lobes, and the superior frontal and parietal cortices. The default mode network has an important role in the representation of a person’s mental state and “internal mentation”, i.e., the introspective mental activities spontaneously emanated by the human brain (Andrews-Hanna, 2012). Such “self-referential thoughts” are necessary to perceive the inner bodily or mental states—including pain and depression.

Self-referential processes have been repeatedly shown to be abnormal, and self-focus is increased in people suffering from depression (Lemogne et al., 2012). Moreover, it has been shown that increased self-focus in depressed individuals may be a predictor of major depressive episodes and chronic depression (Nolen-Hoeksema, 2000). Grimm and her colleagues demonstrated that abnormally increased negative self-attribution, as a hallmark of increased self-focus in major depressive disorders, might be mediated by abnormal neural activity in subcortical-cortical midline structures linked to the default mode network (Grimm et al., 2009, 2011).

There is increasing evidence that the default mode network has a pivotal role in neuronal activity underlying major depressive disorders (Greicius et al., 2007; Xueling et al., 2012; Zhu et al., 2012; Guo et al., 2013; Wenbin et al., 2013) and also in late-life depression (Alexopoulos et al., 2012; Andreescu et al., 2013) and therefore it could serve as a potential biomarker of depressive disorders. Considering the very high relapse rates of patients after a major depressive episode, Li et al. (2013) hypothesized that abnormal default mode network connectivity might persist even after recovery. They have suggested that default mode network functionally dissociates into two subsystems—connectivity in the posterior sub-network is normalized after antidepressant treatment, whereas there was persistent abnormal connectivity in the anterior sub-network.

The above mentioned observations are in accordance with the findings of Marchetti et al. (2012) who propose specific imbalances in the default mode system which could represent a residual neural “depressive scar” that was affected by the severity of previous depressive episodes; it was also suggested that it could be used as a predictor of future depressive episodes.

Chronic pain is associated with changes in brain physiology and anatomy. It has been suggested that neuronal activity underlying subjective perception of chronic pain may be divergent from the activity associated with acute pain. Some studies have indicated that chronic pain can also affect cortical areas unrelated to pain (Apkarian, 2008; Cauda et al., 2012; Hashmi et al., 2013).

Prolonged experience with chronic pain represents a form of emotional learning, shifting from sensory to hedonic neuronal circuits (Farmer et al., 2012). Chronic pain is often accompanied by cognitive and behavioral impairment and decreased quality of life. Increased anxiety, depression and sleep disruption are manifested as an affective association of chronic pain. Moreover, clinicians often observe that these additional effects deepen the patient’s suffering and the effects persist even after the pain is reduced by therapy and the source of nociceptive activity has disappeared (Mansour et al., 2013).

Various studies emphasize the complexity of chronic-pain processes that affect large circuits and stimulate extensive reorganization of cortical function and structure (Apkarian et al., 2009; Apkarian, 2011).

Some authors propose that the structural impairments that accompany chronic pain can also influence functions of the default mode network. Baliki and his colleagues demonstrated that patients suffering from chronic back pain displayed reduced deactivation of various default mode network regions during a simple visual attention task, even though the performance of the patient group and control were the same (Baliki et al., 2008). Similar results were obtained in studies by Tagliazucchi et al. (2010). It is important to point out that these studies demonstrated that alterations of the default mode system, in chronic-pain patients, might influence brain mechanisms responsible for processing information unrelated to pain.

Napadow et al. (2010) demonstrated alternating levels of intrinsic connectivity within multiple brain networks in fibromyalgia, which is a central chronic pain syndrome associated with widespread and spontaneously fluctuating pain. This study showed greater connectivity in the default mode network and in the right executive attention network (in contrast to the default mode network, in which the fronto-parietal executive attention network is involved in cognitive processing associated with working memory and attention). It suggests that fibromyalgia pain might be mediated by alternating activity levels in the central nervous system (CNS) (hyperexcitability) more than by peripheral pathological sensations.

A study by Loggia et al. (2013) revealed that greater clinical pain in patients with chronic low back pain at baseline was associated with greater connectivity between the default mode network and insula (brain region involved in pain processing) and decreased connectivity between the default mode system and the pregenual anterior cingulate cortex (involved in brain inhibition). Moreover, baseline pain correlates positively with the level of connectivity between the default mode network and the right insula; while increased clinical pain, induced by physical maneuvers, is correlated with changes in this connectivity. These results suggest that resting default mode connectivity may also encode the severity of clinical pain.

More and more evidence indicates that chronic pain can, at some point, become a sensation that is spontaneous and independent on any external stimuli. Therefore, the default-mode-network perspective could offer fresh insights into the study of chronic pain. Moreover, since the default mode network is deeply involved in self-referential processes and subjective experience, it could represent a nodal point that is common for both chronic pain and depression.

## Neuroplasticity

Neuroplasticity involves molecular, cellular and synaptic processes that modify connectivity between neurons and neuronal circuits. They are modulated by behavioral, sensory, cognitive and emotional experience, and are also influenced by pathological states and chronic pain or depression. It is important to stress that transient, but repetitive functional changes induced by pain or depression can lead to more permanent changes. Accordingly, long-lasting interference with the normal activity of the default mode network could initiate plastic changes that could lead to irreversible structural and functional changes of the default mode network.

Patients suffering from serious disorders are under chronic stress, associated with a loss or change in social status, loss of positive expectations, feelings of discomfort, etc. This emotional situation induces a spiral of complaints and stresses mediated by neuronal changes which in turn can lead to alterations in other brain functions and structure. Stress is a common biological denominator connecting patient suffering on the one hand, and emotion, cognition and neuroplastic substrates on the other hand. Stress profoundly affects synaptic form and function (Popoli et al., 2011; Sandi, 2011). Stress is also a well-accepted etiological factor in depression.

Stress induces the release of glucocorticoids (GC) that significantly impact hippocampal functions with the potential to enhance or suppress neuroplastic processes. Stress gives rise, by means of the limbic system and activation of the reticular formation, to increased production of corticotropin-releasing hormone (CRH). A feedback loop from the periphery is maintained by the inhibitory effects of GC on CRH production. This feedback involves several types of glucocorticoid receptors, located mostly inside the hippocampus. Overproduction of CRH leads to overproduction of ACTH and, later on, to overproduction of GC. Such a situation occurs after a hippocampal lesion or during chronic stress. Stress also leads to a reduction in brain derived neurotrophic factor (BDNF)—one of the most predominant neurotrophic factors in the adult brain—in the hippocampus and increases it in the amygdala (Höschl and Hajek, 2001; Finsterwald and Alberini, 2013; Hayley and Litteljohn, 2013). While there is strong evidence linking BDNF to stress and depression, other neuronal growth factors are also involved, most notably glial cell-line derived neurotrophic factor (GDNF) and nerve growth factor (NGF; Hayley and Litteljohn, 2013).

Stress modulation of synaptic plasticity is mediated via activation of mineralocorticoids and GC receptors and exert direct effects on neurons and glia cells and also increase glutamate release in the prefrontal cortex, hippocampus and amygdala (Sandi, 2011). It has been shown that elevated levels of corticoids influence learning processes (Bodnoff et al., 1995). Stress events also disrupt long term potentiation in the hippocampus (Shors et al., 1997), which is a key structure for declarative memory (Hölscher, 1999). Pre-clinical and clinical studies have demonstrated that stress and depression can lead to reductions in the total volume of the adult hippocampus. These structural changes may not necessarily be permanent. The degree of volume reduction can provide information regarding treatment effectiveness or response to treatment (Arnone et al., 2013; Hayley and Litteljohn, 2013).

Repeated stress also produces alterations in brain plasticity in animal models; however, the relevance of hippocampal changes to behavioral changes is still matter of debate. For example, the granule cells of the dentate gyrus are significantly affected via a decreased rate of neurogenesis following prolonged stress (Radley and Morrison, 2005). In contrast, chronic antidepressant treatment up-regulates hippocampal neurogenesis, and therefore could block or reverse the atrophy and damage caused by stress. Some studies have also demonstrated that neurogenesis is required for the actions of antidepressants in behavioral models of depression (Warner-Schmidt and Duman, 2006).

The hippocampus is a target for the effects of GCs and stress, which in turn, could influence its ability to regulate the HPA axis. Chronic GC administration at artificially high levels induces apical dendritic retraction and debranching in rat CA3c pyramidal neurons (Woolley et al., 1990; Watanabe et al., 1992), while longer exposure to GC results in more substantial hippocampal damage, such as neuron death, gliosis, and atrophied perikarya in the principal layers, most notably in the CA3c region (Sapolsky, 1985). Repeated stress exerts effects similar to GCs on dendritic remodeling in the CA3. One key feature of prolonged stress is the change in dendritic spine number and morphology of hippocampal formation (medial prefrontal cortex). Such structural synaptic changes may be compensatory in response to glutamatergic and calcium-induced toxicity in these neurons during prolonged periods of stress. Since repeated stress also induces apical dendritic retraction in the CA3, this could have significant consequences for the total synaptic population. In contrast, antidepressants oppose dendrite atrophy and increase apoptosis markers induced by stress in the hippocampus (Silva et al., 2008).

Corticoids affect various neurotransmission systems. They potentiate efflux and inhibit re-uptake of glutamate and increase N-methyl-D-aspartate (NMDA) receptor expression. Furthermore, they decrease expression of neurotrophic factors (Smith et al., 1995) and decrease activity of the GABA-ergic system. Glutamate influenced activity of NMDA receptors and the concomitant decrease in GABA-ergic inhibition leads to calcium influx, followed by depolymerization of cytoskeletal proteins, autolysis and eventually neuronal death (Höschl and Hajek, 2001).

With regard to stress induced structural and functional changes in the hippocampus, in particular reduced hippocampal volume, recent studies have indicated reductions in neurogenesis as well as changes in glial density and reductions in the complexity of dendritic arbors that participate in the volumetric decrease (Hayley and Litteljohn, 2013). Alterations in neurobiological properties can result in faulty communication between the hippocampus, amygdala and cortex, which gives rise to disturbed processes of emotionality (Carballedo et al., 2011). However, future studies are needed to assess the potential contribution of volumetric changes in default mode.

The negative effects of stress on hippocampal synaptic plasticity can be reversed by GC antagonists and monoamine antidepressants (Holderbach et al., 2007). Chronic stress can also affect the expression of AMPA and NMDA subunits and various synaptic proteins (Silva et al., 2008), while antidepressant treatment opposes these changes (Martínez-Turrillas et al., 2007; Barbon et al., 2011). Chronic stress promotes pyramidal dendrite retraction in the medial prefrontal cortex by the mechanism of NMDA receptors (Martin and Wellman, 2011). Additionally, tianeptine, an selective serotonin reuptake inhibitor (SSRI) drug with unexplained antidepressive effects, modulates NMDA receptor function in the hippocampus (Kole et al., 2002). Similarly, some studies point to the antidepressive effects of NMDA antagonists (Berman et al., 2000; Zarate et al., 2006; Li et al., 2010). In addition, monoamine systems that represent typical targets for antidepressants are also required for plasticity modulation under stress.

While an obvious logical link between synaptic plasticity and cognition exists, less well understood is the potential for altered synaptic plasticity to disrupt emotional memory, which may be relevant regarding mood disorders. Regardless, the prefrontal cortex-hippocampus-amygdala circuits are likely dysfunctional in depression (Marsden, 2013). It has been suggested that this leads to decreased cognitive control of emotion, resulting in persistent negative emotional reactivity (Murrough et al., 2011).

Stress-induced neurobiological cascades could represent a critical common pathway underlying the biological and psychological characteristics of the default mode of patients suffering from serious disorders. The neuroplasticity hypothesis of depression shows decreased synaptic plasticity in hippocampal circuits and elevated synaptic plasticity in emotional networks including the amygdala (Nissen et al., 2010). Moreover, reduced hippocampal volume may correlate with impairment of cognitive functions in patients with a major depressive disorder (Frodl et al., 2006). There is a huge body of evidence demonstrating the neuropsychological and cognitive deficits associated with depressive disorders. Such deficits have been found in various areas including attention, information processing, memory, verbal fluency, executive functions and psychomotor speed (for review see Austin et al., 2001; Castaneda et al., 2008; Lee et al., 2012).

Although the clinically beneficial effects of antidepressants are well known their direct impact on cognitive (intellectual and psychomotor) functions in less understood. The clinical effects of antidepressants on cognitive functions, both in healthy volunteers and in patients, were reviewed in papers by Amado-Boccara et al. (1995) and Gorenstein et al. (2006). In 2008, Monleon et al. extensively reviewed the effect of antidepressants on memory in animal models. When assessing the cognitive effect of antidepressants in subjects suffering from depression, it is, in theory, necessary to separate the specific effects on cognition from overall clinical improvement. Another methodological difficulty may be discrepancies between subjective assessments of one’s own state and results from neuropsychological testing (Amado-Boccara et al., 1995).

Monleón et al. (2008) proposed that memory traces should be understood not only as an individual experiences, but also as genetic and epigenetic phenomena. From this point of view, each neural system has its own memory and antidepressants can affect each of these systems. Antidepressants may promote new memories (new neuronal patterns) at the same time that they impair older ones (Monleón et al., 2008).

Strong arguments for the role of antidepressants in promotion of new “memory” traces, through neurogenesis, suggest the role of neurotrophic factors in the etiology of depression and its treatment. Both acute and chronic stress decrease levels of BDNF expression in the hippocampus and conversely, chronic (but not acute) administration of most classes of antidepressants increase BDNF expression in the hippocampus (Nestler et al., 2002).

The pathophysiology of major depressive disorders could also involve GDNF, which plays a role in the development and function of hippocampal cells. GDNF is a neurotrophic factor in the transforming growth factor-b-family (Michel et al., 2008; Wang et al., 2011).

Increasing numbers of studies have demonstrated the significant role of neurotrophic factors in the transmission of both of physiologic and pathologic pain. Neurotrophins (including BDNF and NGF) can act as a pathogenic pain mediator and are known to be increased in several painful conditions. When administered, they lead to pronounced mechanical and thermal hyperalgesia (Obata and Noguchi, 2006; Siniscalco et al., 2011). BDNF is part of synaptic plasticity and central sensitization in a spinal cord. It contributes to the development and continuation of neuropathic pain by activation of NMDA receptors (NR2B-containing NMDA) in the dorsal horn (Geng et al., 2010). Melemedjian et al. (2013) emphasized the role of protein kinases as essential mediators of the maintenance of a centralized chronic pain state. Molecular mechanisms of chronic pain, as with neuronal changes in depressive states, parallel memory engram encoding in the CNS.

However, the above mentioned role of BDNF in depression and chronic pain is even more complicated by the fact that BDNF may have antidepressive or pro-depressive functions, depending on the brain area and circuits (Racagni and Popoli, 2008). Berton et al. (2006) have shown that infusion of BDNF into the nucleus accumbens exerts a pro-depressive-like effect in rodent stress models and blockade of BDNF function in the nucleus accumbens exerts antidepressant-like effects.

A more integrated approach to chronic pain and depression could facilitate a more efficient therapy. For example the animal model of antidepressant activity supports the hypothesis that impaired cognition is an element of depression and treatment with drugs enhancing cognitive performance can help alleviate depression (Knapp et al., 2002). Therapeutic strategies focused on modulation of synaptic plasticity and biological pathways common for stress and pain might prove useful for developing novel treatments for those suffering from cancer pain and associated diseases such as depression.

Emotional distress is significantly higher in patients with chronic pain. Pain and depression, especially associated with tumors, can lead to serious mental and physical stress in patients (Rokyta et al., 2009). Pain is conceptualized as chronic stress and coping strategies play an important role in those experiencing cancer pain. Poor coping strategies may lead to a worsening of pain which will lead to an increase in depression. A recent study, among patients with metastatic breast cancer, found that both pain and obsessing over pain exacerbated depression (Badr and Shen, 2014). On the other hand, patients with lung cancer using a repressive coping style (an effort to inhibit negative feelings through an overly positive view of life) reported lower pain intensity and lower levels of depression (Prasertsri et al., 2011). In the short-term point, this technique may represent a convenient strategy, but for the long-term, repressive coping is not an efficient mood-regulation strategy since it can intensify anxiety dysfunction (Geraerts et al., 2012).

Pain suffering is closely related to psycho-neuro-immunological changes (Rittner et al., 2008); treatment with antidepressants has been shown to normalize immune parameters (Neveu and Castanon, 1999; Rokyta et al., 2009). This integrated treatment could also help increase the patient’s overall quality of life, going beyond the specific clinical target.

## Common neurobiological mechanisms of pain and depression

Pro-inflammatory cytokines such as IL-6, IL-1β and TNF-α may directly modulate neuronal activity in the peripheral and CNS (Ozaktay et al., 2006). Proinflammatory cytokines modulate hippocampal neurogenesis and therefore they can affect the mood. An increased production of proinflammatory cytokines has repeatedly been described in depressive patients (Maes et al., 1997; Connor and Leonard, 1998; Müller, 2013; Bai et al., 2014). Cytokines such as IL-1β, TNF-α and IFN-γ seem to contribute to the pathophysiology of depression by stimulating the hypothalamic-pituitary-adrenocortical axis (Rosenblat et al., 2014), thus activating monoamine reuptake (Raison et al., 2009), and decreasing production of serotonin through increased activity of indoleamine-2,3-dioxygenase (IDO; Müller and Schwarz, 2007).

A meta-analysis performed on patients meeting the DSM criteria for major depression has shown higher concentrations of the proinflammatory cytokines TNF-α and IL-6 in depressed subjects compared to control subjects (Dowlati et al., 2010). Similar associations between depression and C-reactive protein (CRP), IL-6, and, to a lesser extent, IL-1 have been found in patients with cardiac disease or cancer (Howren et al., 2009). A recent study by Breitbart et al. (2014) demonstrated an association between depression and IL-6, but not with other cytokines, in patients with pancreatic cancer. Moreover, IL-6 was not significantly associated with other measures of psychological distress (anxiety and hopelessness) or with symptoms of distress (pain, fatigue, and sleep quality).

Evidence supports the possibility that peripheral inflammatory responses manifest themselves in the CNS in a process known as neuro-inflammation. Therefore the treatment of depression with anti-inflammatory drugs looks like a promising way of targeting more mechanisms. Two individual studies (Müller et al., 2006; Nery et al., 2008) and one meta-analysis (Na et al., 2014) have shown that adjunctive celecoxib combined with antidepressants produced a rapid-onset antidepressant effect and was more effective than placebo combined with antidepressants.

In the pathophysiology of pain, cytokines cause hyperalgesia, reduce the pain threshold, sensitize afferent nociceptive neurons and increase the frequency of discharges in nociceptive A-delta and C fibers. All these factors contribute to central sensitization, which is manifested by secondary hyperalgesia and/or allodynia (Zhang and An, 2007).

In inflammatory pain, IL-1β increases the cyclooxygenase-2 (COX-2) dependent production of prostaglandin E_2_ (PGE_2_), calcitonin gene-related peptide (CGRP; Samad et al., 2001; Neeb et al., 2011) and substance P (Jeanjean et al., 1995), all are factors that induce hypersensitivity. On the other hand, experimental results demonstrate that the neuropeptides substance P and CGRP induce nociceptive sensitization by enhancing IL-1β production in keratinocytes (Shi et al., 2011; Wei et al., 2012).

Celecoxib was the first COX inhibitor with well-defined COX-2 specificity (Tindall, 1999). In animal studies it has been shown that inhibition of spinal COX-2 not only reduces prostaglandin production but also endocannabinoid breakdown (Telleria-Diaz et al., 2010) and expression of purinergic P2X_3_ receptors in the dorsal root ganglia (Wang et al., 2010). These results provide evidence that the pain suppressive effects of COX-2 inhibitors may be mediated either by the endocannabinoid system or by down-regulation of receptors for ATP or both.

Neurotransmitter systems that are used to control pain overlap with those which are considered to be the main pathophysiological mechanisms in depressive disorders, i.e., serotonergic, noradrenergic, and glutamatergic systems.

It has been repeatedly demonstrated, in depressive patients, that there are decreased levels of serotonin metabolites in the cerebrospinal fluid, particularly in patients after suicide attempts (Asberg et al., 1976; Jokinen et al., 2009; Chatzittofis et al., 2013). Tryptophan depletion (a method of lowering brain serotonin, used as a model of depressive disorders) not only worsens depressive symptoms (Fields et al., 1991; Booij et al., 2005; van Steenbergen et al., 2012) and also can increase the sensation of pain (Schwarz et al., 2003; Supornsilpchai et al., 2006; Wei et al., 2010). The system of antinociception operates primarily through serotonin 5-HT1A and 5-HT2 receptors; the stimulation of 5-HT3 receptors, which are found in the periphery, has pronociceptive effects (Campbell et al., 2003).

However, conflicting results follow from both experimental and clinical observations after identical intravenous routes of application of similar doses of 5-HT3 antagonists. While tropisetron, studied in a human model of acute pain induced by intracutaneous electrical stimulation, led to significant analgesic effects (Bandschapp et al., 2011); ondansetron was ineffective in suppression of mechanical allodynia and spontaneous ongoing pain in peripheral neuropathy (Scott et al., 2006; Tuveson et al., 2011).

The SSRI, fluoxetine, which is widely used in the treatment of depression, also has anti-inflammatory properties. Experimental data have shown that fluoxetine inhibits lipopolysaccharide-induced release of nitric oxide (NO) and PGE_2_ in murine serum (Su et al., 2012) and NO and PGE_2_ production by connective tissue cells (Yaron et al., 1999).

Although SSRIs are less potent for treatment of chronic pain, paroxetine, due to its inhibitory effect on P2X_4_ receptors, was found to be effective in suppressing tactile allodynia in a neuropathic pain model in rats (Nagata et al., 2009).

Dysfunction of the noradrenergic system is another pathophysiological characteristic of depression. Noradrenaline levels are decreased, especially in patients who react positively to treatment with noradrenaline reuptake inhibitors (Booij et al., 2003). Conversely, depletion of noradrenaline can cause a relapse of the disease (Delgado and Moreno, 2000; Ruhé et al., 2007). In connection with pain, the mechanism proposed for the analgesic effect of antidepressants is the strengthening of descending serotonergic and noradrenergic systems of antinociception by inhibiting the re-uptake of serotonin and noradrenalin and increasing their availability in the spinal cord. Many experimental and clinical studies with clonidine or medetomidine have demonstrated that the antinociceptive effects of noradrenaline are mediated by α2–adrenoceptors (Kawasaki et al., 2003; Grosu and Lavand’homme, 2010; Blaudszun et al., 2012).

The effect of some antidepressants, especially in neuropathic pain, can also be mediated via the opioidergic system, however, the opioid system appears to be involved in the mechanism of action of antidepressants that only have anti-hyperalgesic action (clomipramine and milnacipran), but not in those with stronger antinociceptive effects such as duloxetine (Wattiez et al., 2011). In a recent study by Bohren et al. (2013), a novel mechanism of antidepressant action was described. They demonstrated that the peripheral nervous system was essential for the anti-allodynic effects of nortriptyline in animal models of neuropathic pain, and acted peripheral β2-adrenoceptors and local inhibition of TNFα production.

Beside these neurotransmitters, substance P is another molecule which participates in the modulation of pain. Substance P is a mediator of C fibers and contributes to central sensitization, depending on the activity of the noradrenergic system. If noradrenergic neuronal activity or the levels of noradrenaline decrease, the increased release of substance P is expressed as hyperalgesia (Jasmin et al., 2002). Depressed patients also had elevated levels of substance P and its level correlated with the severity of clinical symptoms (Bondy et al., 2003).

The dopaminergic system, in terms of pain, has received much less attention, although stimulants like amphetamine or methamphetamine are highly effective analgesics (Yamamotová et al., 2011; Yamamotová and Šlamberová, 2012). Pain is modulated by D2 receptors, particularly within the mesolimbic dopaminergic system and the nucleus accumbens, hence in the “reward system” of the brain (Franklin, 1998; Altier and Stewart, 1999; Wood, 2006).

Both in chronic pain and depression hyperalgesia can results from central sensitization as a consequence of plastic changes in the nervous system. Activation of glutamate NMDA receptors is an essential step in both initiating and maintaining central sensitization, also called the “wind-up” phenomenon (Latremoliere and Woolf, 2009). A study by Klauenberg et al. (2008) demonstrated, for the first time, a considerably enhanced wind-up ratio in depressive patients that was independent of ongoing pain. Wind-up is a physiological process in the spinal cord mainly caused by temporal summation of C-fiber evoked responses that generate a progressive increase in activity of second-order neurons. Consequently, wind-up is increased in some processes with enhanced spinal cord excitability. Ketamine, the non-competitive NMDA receptor antagonist, prevents the wind-up phenomenon and suppresses not only pain but also depression. It acts rapidly and is effective for treatment-resistant patients (Dowben et al., 2013). A recent study showed that a single intravenous dose of ketamine improved depression in 64% of patients within 24 h of administration (Murrough et al., 2013). However, evaluation of antidepressant response showed that not all patients respond to ketamine treatment and that the duration of the antidepressant effect varies across studies (Browne and Lucki, 2013; Sos et al., 2013; Gálvez et al., 2014).

Although the precise mechanisms underlying its antidepressant effects are not fully known, acute administration of ketamine increases BDNF levels in the rat hippocampus. The increase of hippocampal BDNF levels induced by ketamine might also be necessary to produce a rapid onset of antidepressant action in rats (Hayley and Litteljohn, 2013). It should be noted that ketamine also induces rapid and potent anti-inflammatory effects that can be relevant to its antidepressant potential (Hayley and Litteljohn, 2013).

## Are antidepressants more than adjuvant analgesics?

A number of studies have demonstrated frequent co-occurrences of depression and pain, as well as their additive effects in several domains of quality of life in oncological patients (Kroenke et al., 2010). However, the question remains: How do antidepressants affect chronic pain and what is the mechanism of action? Over the last few years we have performed two pilot studies concerning the efficacy of antidepressant treatment in patients with chronic cancer pain and non-cancer pain (Rokyta et al., 2009). Antidepressants were indicated in both groups of patients either for psychiatric comorbidity (depression) and/or neuropathic pain. None of the patients had been treated with antidepressants before entering the study.

The investigation started with 40 patients; 20 non-oncological patients and 20 oncological patients. The most frequent diagnosis in the non-oncological group was low back pain and failed back surgery syndrome. Oncological patients were diagnosed as follows: breast carcinoma, carcinoma of the prostate gland, urinary bladder and kidneys, orofacial cavity and larynx, uterus, gastrointestinal tract and pancreas, lung cancer and leukemia.

Therapy for both groups of patients consisted, most often, of administration of non-steroidal anti-inflammatory drugs and tramadol. As necessary, the above mentioned drugs were used in combination with: opioids, anti-epileptics, antidepressants (fluoxetine and tricyclic antidepressants).

**Figure 1:**
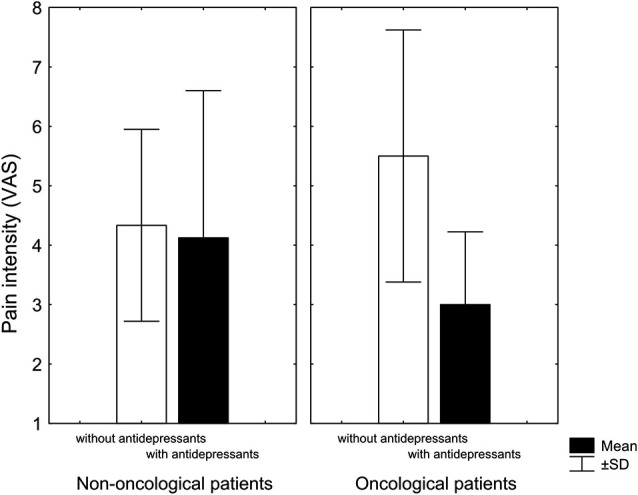
**Pain intensity (visual analogue scale) at the end of treatment of patients with chronic nonmalignant and malignant pain treated with antidepressants (black columns) and patients treated without antidepressants (white columns)**. Antidepressants marginally reduced pain in cancer patients Kruskal-Wallis non-parametric test (*KW-H*_(1,11)_ = 2.9, *p* = 0.08) (KW-H: Kruskal-Wallis non-parametric test) (Adopted from Rokyta et al., 2009).

Although there was no difference in the intensity of pain in non-oncological patients with respect to adjuvant therapy with antidepressants, the surviving oncological patients that used antidepressants reported lower pain intensity than oncological patients not taking antidepressants (Figure 1). Despite the small number of patients, it is interesting that out of 10 patients treated with antidepressants, survived seven, while out of 10 patients not treated with antidepressants, only three patients survived. However, further research with homogeneous diagnostic groups is needed to establish and confirm the observed relationships.

**Figure 2:**
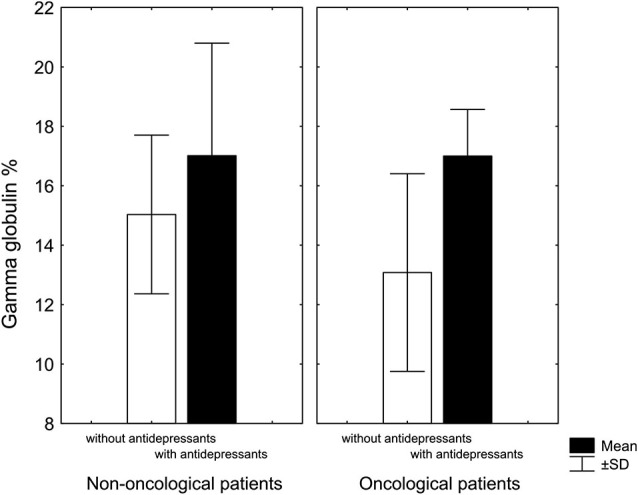
**Gamma globulin levels in patients with chronic nonmalignant and malignant pain associated with antidepressant treatment**. In both groups, patients treated with antidepressants had higher levels of gamma globulin: in non-oncological patients only marginally (*KW-H*_(1,20)_ = 2.7, *p* = 0.09), in oncological patients significantly higher (*KW-H*_(1,18)_ = 7.0, *p* = 0.008) (Adopted from Rokyta et al., 2009).

Another our finding was that chronic pain patients taking antidepressants had, regardless of diagnosis, higher levels of gamma globulin compared to patients not treated with antidepressants (Rokyta et al., 2009; Figure 2). A similar observation was described by Van Hunsel et al. (1996) who followed patients with depression and found, as we did, that depressive patients have low levels of gamma globulin, which rose significantly, after antidepressant treatment. The fact that immune and endocrine systems are closely related in patients with cancer even at the time when patients were informed about their diagnosis was confirmed by a negative correlation between cortisol and CD4 lymphocytes and a positive correlation between cortisol and CD3 lymphocytes. Moreover, patients with a malignant diagnosis had lower cortisol level than patients with a benign diagnosis (Křikava et al., 2007).

Some research observations indicate that depressed patients treated with antidepressants undergo a normalization of immune parameters (Neveu and Castanon, 1999). Normalization of serum cortisol was shown in patients with severe chronic pain treated with opioids (Tenant and Hermann, 2002). From these clinical studies, it is not possible to conclude whether antidepressants and/or opioids have a direct effect on the immune and endocrine system or whether their supposed effects resulted from improved mood.

Opioid peptides are found in many leukocyte subpopulations including lymphocytes, monocytes, and granulocytes circulating in the peripheral blood. Neurokinin substance P is one of many factors that influence migration of opioid-containing leukocytes. NK1 receptor antagonists seem to act peripherally by directly inhibiting the recruitment of opioid containing leukocytes to sites of inflammation (Rittner et al., 2008). Although opioids are frequently used for the treatment of severe pain in patients with cancer, chronic morphine treatment can also have serious negative effects on tumor growth. Morphine stimulates angiogenesis-dependent tumor growth via stimulation of endothelial NO and COX-2 production (Gupta et al., 2002). Administration of celecoxib together with morphine in murine breast cancer model not only prevented promotion of angiogenesis, tumor growth, metastasis and mortality but also led to better analgesia than with morphine or celecoxib alone (Farooqui et al., 2007). Similar potential therapeutic effects were observed for lumiracoxib by Fox et al. (2004) in a model of bone cancer pain in rats, which were attributed to its anti-hyperalgesic activity.

Other antidepressants have also been studied in animal models of cancer. For example, Fang et al. (2012) found that *in vivo* chronic mirtazapine treatment inhibited tumor growth and prolonged the survival of colon carcinoma-bearing mice. The IFN-γ levels in tumors of mice treated with mirtazapine were significantly higher, while TNF-α expression was lower than in untreated mice.

On the other hand, antidepressant pretreatment with desipramine or fluoxetine increased metastasis formation in mice with melanoma, shortened survival, decreased splenocyte anti-tumor natural killer cell cytotoxicity (*in vitro*), and IFN-γ production (Kubera et al., 2011).

One question arising from our study concerns whether the higher mortality seen in the tumor pain patients without antidepressants was a coincidence or whether it suggested some protective function associated with antidepressants. Meta-analyses from human and animal studies have concluded that several antidepressants have a significant positive association with cancer protection, while others have shown a negative association; the effect seems to be dependent on the type of cancer and the type of antidepressant (Steingart and Cotterchio, 1995; Lussier et al., 2004; Walker et al., 2011, 2012; Bielecka and Obuchowicz, 2013; Jahchan et al., 2013).

Knowledge regarding the role of antidepressants in cancer progression or suppression is essential for choosing the proper treatment and clinicians who wish to use antidepressants in cancer treatment need to take into consideration the type of antidepressant, type of tumor, type of anticancer therapy, as well as the patient’s age, phase of cancer and others factors (Bielecka and Obuchowicz, 2013).

It is not possible to unambiguously declare that only one source of the depressive state in oncological patient has a direct relation between depression and pain. There are many different aspects to oncological diseases and their treatment. Depressive states may be caused not only by pain, but also by decreased quality of life, worsening of cognitive functions (Baudino et al., 2012), difficulties accompanying oncological treatment such as gastrointestinal distress and fatigue, and poor life perspectives.

However, we assume that both depression and pain, even though they are experienced in highly subjective ways, are deeply grounded in the neuronal and physiological substrate and therefore can, even if only indirectly, interact on this basis. Chronic pain may alter different systems, including the emotional state and gradually lead to depression, conversely depression affected cognition and perception and may lead to pain sensitization (Torta and Munari, 2010).

## Conclusion

Our working hypothesis supposes that depression and chronic pain produce common negative neuroplastic changes in the CNS. The positive impact of antidepressants would result in a reduction of these pathological cellular/molecular processes and in the amelioration of symptoms, but it may also increase survival times and quality of life of patients with chronic cancer pain. The benefits go beyond prolongation of lifespan because they are also linked to an improvement in the quality of life of treated patients. These effects represent the most important aspects of antidepressant treatment. After careful validation of both experimental and clinical results, this approach could be ready for clinical practice in a relatively short time, especially in oncology, algesiology and psychiatry.

## Author contributions

Richard Rokyta, Karel Vales, Anna Yamamotova and Tereza Nekovarova made substantial contributions to the conception of the paper and are co-responsible for formulation of the hypothesis. Karel Vales contributed mainly to the part of the manuscript concerning neuroplasticity, Anna Yamamotova, Richard Rokyta and Jitka Fricova contributed mainly to the part of the manuscript concerning neurobiological substrate of pain and depression and to the part concerning mechanisms of antidepressants. Tereza Nekovarova contributed mainly to the part focusing on default mode network. Ales Stuchlik critically reviewed the manuscript.

## Conflict of interest statement

The authors declare that the research was conducted in the absence of any commercial or financial relationships that could be construed as a potential conflict of interest.
